# Performance of retail pharmacies in low- and middle-income Asian settings: a systematic review

**DOI:** 10.1093/heapol/czw007

**Published:** 2016-03-08

**Authors:** Rosalind Miller, Catherine Goodman

**Affiliations:** Department of Global Health and Development, London School of Hygiene and Tropical Medicine, London, UK

**Keywords:** Asia, developing countries, pharmacies, private sector, quality of care

## Abstract

In low- and middle-income countries (LMIC) in Asia, pharmacies are often patients’ first point of contact with the health care system and their preferred channel for purchasing medicines. Unfortunately, pharmacy practice in these settings has been characterized by deficient knowledge and inappropriate treatment. This paper systematically reviews both the performance of all types of pharmacies and drug stores across Asia’s LMIC, and the determinants of poor practice, in order to reflect on how this could best be addressed. Poor pharmacy practice in Asia appears to have persisted over the past 30 years. We identify a set of inadequacies that occur at key moments throughout the pharmacy encounter, including: insufficient history taking; lack of referral of patients who require medical attention; illegal sale of a wide range of prescription only medicines without a prescription; sale of medicines that are either clinically inappropriate and/or in doses that are outside of the therapeutic range; sale of incomplete courses of antibiotics; and limited provision of information and counselling. In terms of determinants of poor practice, first knowledge was found to be necessary but not sufficient to ensure correct management of patients presenting at the pharmacy. This is evidenced by large discrepancies between stated and actual practice; little difference in the treatment behaviour of less and more qualified personnel and the failure of training programmes to improve practice to a satisfactory level. Second, we identified a number of profit maximizing strategies employed by pharmacy staff that can be linked to poor practices. Finally, whilst the research is relatively sparse, the regulatory environment appears to play an important role in shaping behaviour. Future efforts to improve the situation may yield more success than historical attempts, which have tended to concentrate on education, if they address the profit incentives faced by pharmacy personnel and the regulatory system.

Key Messages

- Pharmacy practice in Asia is characterised by insufficient history taking; a lack of appropriate patient referral; poor adherence to treatment guidelines, inappropriate supply of medicines and insufficient counselling.
- Adequate knowledge alone is not sufficient to ensure appropriate management of patients presenting at the pharmacy. Profit incentives and the regulatory environment must be taken into consideration when designing interventions to improve pharmacy practice in these settings.
- Intervention research in this area appears to be lacking and more research is particularly required on non pharmacist-run pharmacies and unregistered drug shops.

## Introduction

The role of the private sector in the provision of medicines has traditionally been neglected by governments and the international public health community alike (Bigdeli *et al.* 2014). Yet in most low-and middle-income countries (LMIC) it is widely established that private pharmacies and drug stores are typically patients’ first point of contact with the healthcare system and the preferred channel through which to purchase medicines (Smith 2009b). For example, in Asia, pharmacies have been found to be the dominant source of healthcare for all common problems amongst poor populations in Bangladesh (Khan *et al.* 2012) and in Western Nepal, amongst mothers seeking care for their children, pharmacies were the most popular source (46.2%) (Sreeramareddy *et al.* 2006). Their appeal lies in long opening hours, availability of medicines (including the possibility of credit and the option to purchase medicines in small quantities), geographic accessibility and personal familiarity (Van Der Geest 1982; Logan 1983; Kloos *et al.* 1986, Greenhalgh 1987; Igun 1987; Haak 1988; Price 1989; Mayhew *et al.* 2001). Further, many patients have neither the time nor money to consult a physician (Ferguson 1981; Wolffers 1987; Haak 1988; Saradamma *et al.* 2000; Seeberg 2012). These drug shops range from high end outlets staffed by pharmacists, to small, rural, roadside stalls staffed by someone without formal health qualifications. Unfortunately, it is all too common that drug selling at these outlets meets the World Health Organization’s (WHO) criteria for being ‘irrational’. That is, patients do not receive the appropriate medicines, in doses that meet their individual requirements, for an adequate duration, and at the lowest cost (Holloway and Van Dijk 2011). To develop appropriate interventions to address this, it is first necessary to understand the nature of the problem and also the determinants of provider behaviour to reflect on how this could best be changed.

Many papers have reported on the inadequacies of pharmacy practice in LMIC, and some reviews have been conducted (Smith 2009b; Wafula *et al.* 2012). However, no up to date systematic review is available on performance of pharmacies and drug stores across Asia’s LMIC. This article aims to address that gap, and in addition, present the first systematic review of the determinants of poor practice in these settings, substantially updating and expanding previous reviews in this area (Goel *et al.* 1996; Radyowijati and Haak 2003).

## Methods

### Scope of review

In many LMIC, there are a wide range of outlets selling medicines to their local communities. This review is concerned with the full range of establishments whose primary business is selling medicines. Papers reporting on pharmacist-run pharmacies (PRPs), non pharmacist-run pharmacies (NPRPs) and both registered and non-registered outlets were eligible for inclusion. Despite the legal requirement to have a trained pharmacist on duty at all times, in reality, in many countries in Asia, these pharmacies are typically operated by staff who are not authorized to do so (Wolffers 1987; Kamat and Nichter 1998; Chuc and Tomson 1999; Seeberg 2012; Vu *et al.* 2012). In countries where a shortage of pharmacists exists, NPRPs are permitted; these are registered outlets but the presence of a graduate pharmacist is not mandatory. These pharmacies are typically staffed by personnel with limited medical training and are sometimes restricted in the repertoire of medicines they are permitted to sell. Unregistered drug shops sell medicines informally and are not legally recognized by the health system of the countries in which they operate (Wafula and Goodman 2010). From this point onwards, ‘pharmacy’ will be used as an umbrella term to denote all types of outlets selling medicines.

The first part of the review is concerned with performance of pharmacies which, in this instance, is conceptualized as behaviour relating to the sale of medicines, either with or without a prescription, or the provision of advice (the importance of the sale of substandard and counterfeit medicines in Asia is also recognized (Cockburn *et al.* 2005; Newton *et al.* 2006; World Health Organization 2006; Institute of Medicines 2013), but considered beyond the scope of this review). The second part of the review concerns the determinants of poor practice, that is, the factors that contribute to practices that are deemed inappropriate.

### Search strategy

A broad search strategy combining MeSH and free text terms was used to search PubMed, Embase, Econlit, PsychInfo, Web of Science, Global Health and International Pharmacy Abstracts with the aim of identifying all studies of pharmacies in LMIC in Asia (World Bank 2014) (see Table 1).

**Table 1.** Search strategy

Target population (combined by ‘OR’)		Geographic location (combined by ‘OR’)	
**Free text terms** Chemist Drug retailer^a^ Drug shop^a^ Drug seller^a^ Drug vendor^a^ Drug store^a^ Drug dispensing Drug outlet^a^ Druggist Medicine retailer^a^ Medicine shop^a^ Medicine seller^a^ Medicine vendor^a^ Medicine store^a^ Medicine dispensing shop^a^ Medicine outlet^a^ Medicine dealer^a^ Pharmacies Pharmacy Pharmacy service^a^ Pharmacist^a^ Pharmaceutical service^a^	**MeSH terms** Pharmacy Pharmacies	**Free text terms** Developing countries Developing country Low-income country Low-income countries Middle-income country Middle-income countries Asia	**MeSH terms** Developing countries Asia

In addition to the aforementioned databases, the International Network for Rational Use of Drugs (INRUD) bibliography and the WHO’s essential medicines and health products information portal were searched.

**Figure 1.**
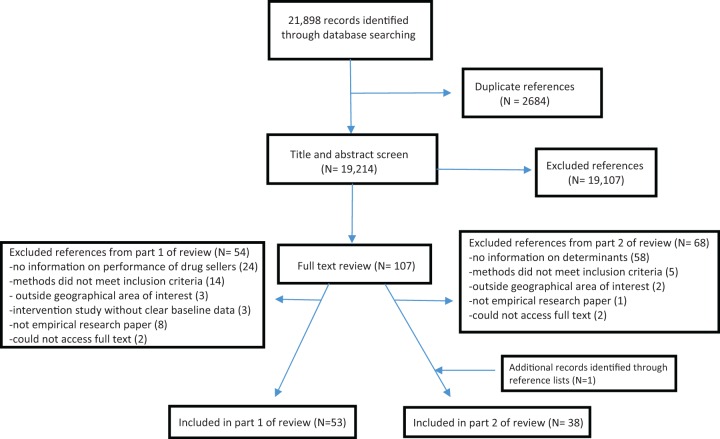
PRISMA flow diagram.

The search was restricted to English language studies published between 1 January 1984 and 29 August 2014. A total of 21 898 papers were initially identified; after removing duplicates, 19 214 titles and abstracts were screened by R.M.; the full-text of 107 records were obtained; 53 met our inclusion criteria for part 1 of the review and 38 met the criteria for part 2 (see Figure 1). Bibliographies of eligible texts were scanned to identify any further relevant papers.

For part one, papers needed to report on performance relating to the sale of medicines, either with or without a prescription, or the provision of advice. This included studies employing both quantitative and qualitative methodologies. In order to collect the most accurate data on pharmacy practice, only studies utilising methods that collected data at the outlet and relied on a third party observation of practice were included (studies relying on self-reported practice were excluded due to the risk of desirability bias and those collecting data on medicine use through household surveys were excluded due to the high risk of recall bias). Intervention studies were included, only where they provided baseline data; this was thought to reflect standard practice. Where baseline data could not be disentangled from post-intervention results, studies were excluded from part one.

Eligible studies for part two reported on both pharmacy performance (applying the same method criteria as for part one) and possible determinants of that reported performance. Where changes in practice could be attributed to an intervention strategy, these intervention studies were included and strategies were viewed as determinants of practice. For example, a training intervention could shed light on the importance of knowledge as a determinant of practice. Additionally, qualitative studies which sought to understand the determinants of practice behaviours were also included. Of the studies included in part two, 31 of these were a subset of papers from part one; three papers were intervention studies where the baseline results were not clear; three papers were intervention studies where the baseline results were described by other papers from the same research study and one paper reported results from a qualitative study solely focussing on determinants of poor practice.

Data from the included studies were extracted into an excel database under the following headings: date and location of study, which aspects of performance measured, sampling and study design, data collection methods, details of intervention, main findings—performance, main findings—determinants of performance. Key emergent themes recurring across the data were discussed between the authors and a narrative synthesis was conducted. See Supplementary Appendix 1 for a full list of included studies and key characteristics.

## Results

### Part one: performance of pharmacies in LMIC in Asia

**Figure 2.**
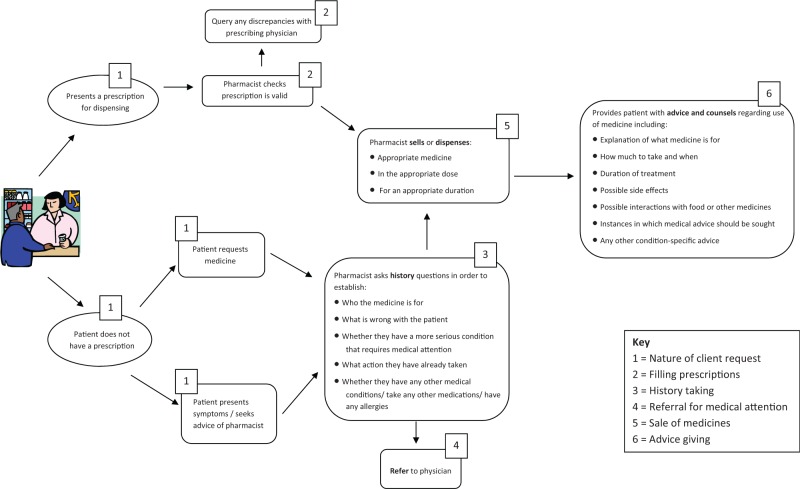
Appropriate retail pharmacy practices for patients with and without a prescription.

This literature review reveals a number of shortcomings in pharmacy practice. We have organized our findings according to the stages of an encounter in the pharmacy (Figure 2). Following an overview of the included studies, we report on six key stages, namely, the nature of requests from patients, filling of prescriptions, history taking, referral for medical attention, sale of medicines and advice giving.

#### Overview of included studies

This part of the review identified a total of 53 papers from 43 studies in 14 countries (some studies collected data in more than one country). Papers coming from the same research project have been grouped together and the term ‘study’ is used to denote distinct pieces of research. The most researched countries were India and Vietnam, with 10 and 9 studies, respectively. Five studies reported on each of Thailand, Bangladesh and Nepal, three on Indonesia, two on Sri Lanka and Pakistan and one on The Philippines, Mongolia, Malaysia, Yemen Arab Republic, Syria and Lao PDR. Bangladesh and Nepal are low-income countries, Thailand and Malaysia are higher-middle and all the others are classified as lower-middle income economies (World Bank 2014).

Studies reported on the full range of pharmacies. PRPs were included in the majority of research projects. Several papers reported on outlets legally entitled to operate without a pharmacist, including type II pharmacies in Thailand, class II and III pharmacies in Lao PDR, type C pharmacies in Pakistan, drug stores in Indonesia and drug retailers in Nepal (Kafle *et al.* 1996; Stenson *et al.* 2001a; Hadi *et al.* 2010; Saengcharoen and Lerkiatbundit 2010;Hussain and Ibrahim 2011). Only one study from Indonesia reported on unregistered outlets (Hadi *et al.* 2010).

Researchers employed a range of methods to collect data on pharmacy practice. Simulated client methodology was used widely (31 studies) to investigate how a range of requests and conditions are managed. These included requests for specific prescription only medicines (POMs), contraceptives and treatment for fever, skin abrasions, diarrhoea, sexually transmitted infections (STIs), respiratory tracts infections (RTIs), tuberculosis, asthma, migraine and anaemia. 12 studies used observation in order to record the details of transactions between pharmacy staff and customers. Exit interviews with patients were used less frequently (seven studies) to gather information about medicines purchased and staff behaviour.

1. Nature of client requestsClients with health concerns visited the pharmacy for three main reasons: to fill a prescription following a medical consultation; to purchase a specific medicine(s) or to seek medical advice from the pharmacy staff. In order to examine staff behaviour and to put certain practices into perspective, it is useful to understand the frequencies of these different types of scenarios. Eight studies observed all transactions in sampled pharmacies for a fixed period of time, ranging from 2 h to 2 weeks per pharmacy. The proportion of transactions where medicines were purchased without a prescription ranged from around half in studies from Pakistan and India (Krishnaswamy *et al.* 1985; Greenhalgh 1987; Kamat and Nichter 1998; Basak and Sathyanarayana 2010; Hussain and Ibrahim 2011), to over 80% in a study from Lao PDR (Chuc and Tomson 1999; Syhakhang *et al.* 2001) and virtually all transactions in studies from Vietnam and Malaysia (Chua *et al.* 2013). Of the medicines purchased without a prescription, three studies reported that around one-third were recommended by the pharmacy staff (Syhakhang *et al.* 2001; Basak and Sathyanarayana 2010; Chua *et al.* 2013). Other studies reported that the vast majority of medicines sold without a prescription were requested by the client, with pharmacists advising on <5% of these purchases (Krishnaswamy *et al.* 1985; Kamat and Nichter 1998; Chuc and Tomson 1999; Hussain and Ibrahim 2011). Only studies from India reported on common ways patients requested medicines. These were by name, on a scrap of paper, or by bringing in an old sample (Greenhalgh 1987; Kamat and Nichter 1998; Saradamma *et al.* 2000). Studies from Vietnam, India and Bangladesh reported that at least half of clients were buying medicines for someone other than themselves, most commonly a family member (Dua *et al.* 1994, Duong *et al.* 1997b; Roy 1997; Kamat and Nichter 1998). One study noted that domestic servants were commonly sent to purchase medications on behalf of their employers (Kamat and Nichter 1998).
2. Filling prescriptionsOnly a few studies made reference to the handling and processing of prescriptions in the pharmacy. Several poor practices were reported. Prescriptions were rarely validated by dispensers (Hussain and Ibrahim 2011), old prescriptions were frequently honoured (at the extreme, patients were seen to be reusing prescriptions 5 years out of date) (Greenhalgh 1987; Kamat and Nichter 1998; Basak and Sathyanarayana 2010), and prescriptions were returned to customers after dispensing for reuse in the future (Puspitasari *et al.* 2011). Further, doctors’ prescriptions were not always dispensed as intended. Some studies indicated that where patients cannot afford to buy all items on a prescription, the pharmacists played an important role in advising patients what they should purchase in light of their financial constraints (Greenhalgh 1987; Roy 1997; Kamat and Nichter 1998). Studies did not report a single example of a pharmacist querying a prescription with the doctor (despite ample description of inappropriate prescribing practices).
3. History takingIn general, history taking in pharmacies was found to be inadequate. The majority of these data come from studies where mystery shoppers presented with symptoms of various conditions, including childhood diarrhoea and STIs. Most questioning was limited to enquiring about the presence of other related symptoms and often this was only observed in a subset of the study pharmacies (Ross-Degnan *et al.* 1996; Chuc *et al.* 2001, Bista *et al.* 2002; Van Sickle 2006; Saengcharoen and Lerkiatbundit 2010; Minh *et al.* 2013). It was rare for pharmacists to ask whether the patient suffered from any other conditions or allergies, took any other medication or had tried any treatments before consulting at the pharmacy (Wachter *et al.* 1999; Chuc *et al.* 2001; Van Sickle 2006; Saengcharoen and Lerkiatbundit 2010; Hussain and Ibrahim 2012). Worryingly, a common finding was that very few, if any, pharmacists enquired about danger signs that would indicate a more serious underlying cause and warrant immediate medical attention (Modal and Lamba 1994; Kafle *et al.* 1996; Duong *et al.* 1997a; Wachter *et al.* 1999; Saengcharoen and Lerkiatbundit 2010; Rathnakar *et al.* 2012). At the extreme end of poor practice, some pharmacists were found to ask not a single question (Duong *et al.* 1997a; Chalker *et al.* 2000; Rathnakar *et al.* 2012).Only three studies measured pharmacists’ responses to direct requests for medications. Questioning was insufficient on who the medicine was for, what symptoms they were experiencing, whether they took any other medication or whether they suffered from drug allergies (Ratanajamit and Chongsuvivatwong 2001; Ratanajamit *et al.* 2002; Larsson *et al.* 2006; Puspitasari *et al.* 2011). Additionally, Kamat and Nichter (1998) describe the ‘quick presentation of tablets and fast exchange of money’.
4. Referral for medical attentionSome studies used simulated clients to present with symptoms of conditions that necessitate referral for medical attention; these included diarrhoea (of 3 day duration) in an 11 month old, cystitis in a man, STIs, asthma and tuberculosis. These conditions require examination, diagnostic testing or POMs and hence their management is outside the remit of a pharmacist’s expertise. Referral practices of pharmacists were found to be unsatisfactory. Tuberculosis was the condition most commonly referred outside the pharmacy—46% of 138 pharmacies in Vietnam, although only 9% of patients were directed to a designated TB facility (Vu *et al.* 2012). For the other conditions, referral proportions ranged from 7% to 37% (Tomson and Sterky 1986; Tuladhar *et al.* 1998; Wachter *et al.* 1999; Chalker *et al.* 2000; Van Sickle 2006; Apisarnthanarak *et al.* 2008).
5. Sale of medicinesIn terms of medicine provision, a number of concerns are raised by the literature regarding the appropriateness of medicines sold (with attention to the legal, clinical and physical aspects of their sale), dosing and duration of treatment.
  - Appropriateness of medicines
    - Legal appropriatenessThe sale of POMs without a prescription was a phenomenon found to be prevalent in all 14 countries reported on. A wide range of medicines, which are only legally permitted to be sold with a prescription, were freely available without a prescription including: antibiotics, steroids, antimalarials, narcotic analgesics, psychotropics, anti-epileptics, antihypertensives, anti-diarrhoels, antihistamines, sedatives, tranquilisers, hypoglycaemic medicines, anti-tuberculosis agents and even, on occasion, abortifacients (Krishnaswamy *et al.* 1985; Tomson and Sterky 1986; Greenhalgh 1987; Wolffers 1987; Lansang *et al.* 1990; Dua *et al.* 1994; Modal and Lamba 1994; Dineshkumar *et al.* 1995; Duong *et al.* 1997a,b; Roy 1997; Cong *et al.* 1998; Kamat and Nichter 1998; Chuc and Tomson 1999; Wachter *et al.* 1999; Saradamma *et al.* 2000; Chuc *et al.* 2002; Larsson *et al.* 2006; Mac *et al.* 2006; Mamun *et al.* 2006; Qidwai *et al.* 2006; Basak and Sathyanarayana 2010; Hadi *et al.* 2010; Nakajima *et al.* 2010; Al-Faham *et al.* 2011; Puspitasari *et al.* 2011; Hussain and Ibrahim 2012; Ngo *et al.* 2012; Rathnakar *et al.* 2012; Seeberg 2012; Vu *et al.* 2012; Huda *et al.* 2014; Nga *et al.* 2014). In Thailand, antibiotics are legally permitted to be sold by class I drug stores but not in lower class stores. This restriction is reportedly not observed in practice (Podhipak *et al.* 1993; Saengcharoen and Lerkiatbundit 2010). In Indonesia, antibiotics were found to be freely available through unregistered roadside kiosks (Hadi *et al.* 2010).
    - Clinical appropriatenessThe majority of the literature paints a dismal picture of treatment practices in pharmacies across Asia. More often than not, medicines that are dispensed are not clinically appropriate for the patients’ needs. Table 2 shows treatment choices by pharmacy staff for a number of conditions in response to visits from simulated clients. At best, studies report that recommended practices are followed in less than half of encounters (Chuc *et al.* 2001; Bista *et al.* 2002). At worst, only a handful, and in some cases no staff were found to follow best practice guidelines (Duong *et al.* 1997a; Van Sickle 2006; Hussain and Ibrahim 2012; Vu *et al.* 2012).

**Table 2.** Treatment choices for a range of conditions presented at pharmacies in Asia by simulated clients

Condition	Countries, number of distinct studies and references	Recommended treatment	Sample percent giving appropriate treatment	Details of inappropriate treatment
Watery diarrhoea (in children and adults)	−Pakistan, Thailand, India, Sri Lanka, Bangladesh, Yemen Arab Republic, Indonesia, Vietnam, Nepal −11 studies −(Tomson and Sterky 1986; Podhipak et al. 1993; Modal and Lamba 1994; Ross-Degnan et al. 1996; Duong et al. 1997a; Wachter et al. 1999; Qidwai et al. 2006; Saengcharoen and Lerkiatbundit 2010; Hussain and Ibrahim 2012b; Kafle et al. 2013; Minh et al. 2013; Pham et al. 2013)	International recommendations (WHO) −Oral rehydration salts (ORS) −Zinc supplementation	−Pharmacies recommending ORS ranged from 3% to 45% (Two studies <10%; three studies 10-19%; six studies 20-45%)	−Unnecessary antibiotics were recommended in every study and anti-diarrhoels in nine studies −Pharmacies recommending antibiotics ranged from 2% to 97% and anti-diarrhoeals from 19% to 88% −Other unnecessary medicines recommended were anti-spasmodics and anti-motility drugs
Sexually transmitted infections	−Nepal, Vietnam, Bangladesh −5 studies −(Tuladhar et al. 1998; Wachter et al. 1999; Chalker et al. 2000; Rahman et al. 2000; Bista et al. 2002)	National guidelines (Nepal- not stated for Vietnam or Bangladesh): −Uncomplicated cystitis in men: course of antibiotics^a^ −Gonorrhoea: ciprofloxacin 500 mg stat −Chlamydia: doxycycline 100 mg twice a day (patients with vaginal or urethral discharge in Nepal are to be treated for chlamydia and gonorrhoea)	−Three studies where clients presented with discharge, treated with correct medicines in 34% and 2% of cases in Nepal and 0% in Vietnam −Dysuria (indicative of cystitis) was treated with antibiotics in 38% of cases −0% treated according to national standardised guidelines for urethral discharge or genital ulcer in Bangladesh	−Injection recommended for urethral discharge −Urinary alkalizers commonly sold −Other medicines recommended included vitamins, topical antibiotics, antihistamines and, occasionally, disinfectants
Mild respiratory tract infection in children	−Vietnam −1 study −(Chuc et al. 2001)	National guidelines: (Vietnam) −In absence of danger symptoms advise to treat: -fever with paracetamol −Chesty cough with expectorants −Sore throat and cough with traditional medicines -do not prescribe antibiotics or remedies containing codeine or antihistamine	−36% of advice was in line with national guidelines	−Antibiotics were given in 42% of encounters −83% of pharmacies gave antibiotics in at least one encounter −Antitussives commonly prescribed, of which 40% contained codeine or an antihistamine
Asthma	−India −1 study −(Van Sickle 2006)	International guidelines for mild asthma: − Daily inhaled corticosteroid − Inhaled β_2 -_ agonist for symptom relief when required	−93% of pharmacies dispensed medication; 0% gave either of the recommended inhalers	−Most commonly recommended medicines were oral β_2 -_ agonists, methylxanthines, antibiotics and oral corticosteroids −40 unique combinations of drugs were sold to clients
Tuberculosis	−Vietnam −1 study −(Vu et al. 2012)	National guidelines: −Combination treatment with anti-tuberculosis drugs (isoniazid, rifampicin, pyramzinamide and ethambutol)	−53% of pharmacists dispensed drugs; 0% gave anti-tuberculosis medicines	−Details of drugs dispensed not given
Pregnancy-related anaemia	−Nepal −2 studies −(Kafle et al. 1996, 2013)	International recommendations: −Simple iron-folate combination −Ferrous sulphate	−1% of pharmacies gave iron-folate alone and none gave ferrous sulphate −58–71% gave an iron preparation of some kind (whilst not the recommended product, still clinically effective, but more costly)	−12% gave a vitamin or mineral product that was not therapeutic for pregnancy-related anaemia −9% dispensed tonics that were not therapeutic
Migraine	−Thailand −1 study −(Saengcharoen and Lerkiatbundit 2013)	International recommendations: −NSAID for mild migraine −Ergotamine for moderate migraine	−33% dispensed appropriately for mild migraine −54% dispensed appropriate medicine for moderate attack	−Inappropriate prophylactic medicines such as propranolol and atenolol were recommended by 28% and 18% for moderate and mild attacks respectively

^a^Details of specific antibiotics unspecified by study.

These studies also reveal that a number of inappropriate treatments are given, either in addition to, or, instead of recommended treatments. Some of the medicines, such as tonics for anaemia, have no therapeutic value (Kafle *et al.* 1996). Others can be harmful, for example the use of anti-diarrhoels and antitussives in infants and children (Tomson and Sterky 1986; Podhipak *et al.* 1993; Modal and Lamba 1994; Ross-Degnan *et al.* 1996; Duong *et al.* 1997a; Chuc *et al.* 2001; Qidwai *et al.* 2006; Saengcharoen and Lerkiatbundit 2010; Hussain and Ibrahim 2012; Minh *et al.* 2013).Another concern is the overuse of antibiotics. A number of papers, report that antibiotics were commonly sold for young babies through to adults for a host of symptoms and conditions for which they are not indicated, including: diarrhoea, asthma, upset stomachs, coughs, colds, runny noses, influenza and (non-infected) skin abrasions (Tomson and Sterky 1986; Thamlikitkul 1988; Podhipak *et al.* 1993; Modal and Lamba 1994; Ross-Degnan *et al.* 1996; Duong *et al.* 1997a,b; Wachter *et al.* 1999; Chuc *et al.* 2001; Chuc *et al.* 2002; Qidwai *et al.* 2006; Van Sickle 2006; Apisarnthanarak *et al.* 2008; Saengcharoen and Lerkiatbundit 2010; Hussain and Ibrahim 2012; Minh *et al.* 2013). Further, even where antibiotics are indicated, inappropriate choices were made (Tuladhar *et al.* 1998; Bista *et al.* 2002). In studies from the Philippines, India, Vietnam and Lao PDR antibiotics were found to make up between a fifth and a third of all purchases (Lansang *et al.* 1990; Dua *et al.* 1994; Syhakhang *et al.* 2001; Basak and Sathyanarayana 2010; Nga *et al.* 2014). Tetracycline was reportedly sold to children (for whom it is contraindicated) in studies from Vietnam, India and Thailand (Thamlikitkul 1988; Dua *et al.* 1994; Chuc and Tomson 1999). Further, the widespread use of chloramphenicol for minor conditions is a concern due to the risk of bone marrow suppression (Greenhalgh 1987; Thamlikitkul 1988; Syhakhang *et al.* 2001).Vitamins, tonics and nutritional products with dubious pharmacological value were found to constitute a significant proportion of medication costs in studies from India, Bangladesh, Vietnam and Nepal (Krishnaswamy *et al.* 1985; Greenhalgh 1987; Dineshkumar *et al.* 1995; Kafle *et al.* 1996; Duong *et al.* 1997b; Roy 1997; Chuc and Tomson 1999; Saradamma *et al.* 2000; Basak and Sathyanarayana 2010). They were often seen as an essential accompaniment to antibiotics (Greenhalgh 1987; Dua *et al.* 1994; Roy 1997; Saradamma *et al.* 2000). A final observation was the popularity of (often irrational) combination products (Tomson and Sterky 1986; Greenhalgh 1987; Lansang *et al.* 1990; Dineshkumar *et al.* 1995; Kafle *et al.* 1996; Chuc and Tomson 1999; Wachter *et al.* 1999; Saengcharoen and Lerkiatbundit 2010).
    - Physical appropriatenessFew studies investigated or commented on the physical state of the medicines sold to patients. Those that did, reported that drugs were often sold in loose strips, as opposed to in their original package; they were rarely labelled; and different medicines were mixed together in the same package (Kamat and Nichter 1998; Stenson *et al.* 2001a; Van Sickle 2006; Hadi *et al.* 2010; Hussain and Ibrahim 2011). As a result, information regarding dose, expiry date and active ingredients was not clear. In six of 28 shops visited in Sri Lanka, tetracycline was not stored in a refrigerator as it should be (Wolffers 1987).
  - Appropriateness of dose and duration of treatmentSeveral studies report that medicine dosing was outside of the therapeutic range for a variety of conditions (both sub-therapeutic and over dose) (Thamlikitkul 1988; Tuladhar *et al.* 1998; Rahman *et al.* 2000; Bista *et al.* 2002). Saradamma et al. (2000) study of antibiotic purchases in India found that those without a prescription were three times more likely to purchase an inadequate dose than those with a prescription (66% vs 40%). The problem of incomplete courses of antibiotics was reported in India, Thailand, Vietnam, Bangladesh, The Philippines and Sri Lanka (Tomson and Sterky 1986; Greenhalgh 1987; Lansang *et al.* 1990; Duong *et al.* 1997a,b; Cong *et al.* 1998; Chuc and Tomson 1999; Chalker *et al.* 2000; Saradamma *et al.* 2000; Mamun *et al.* 2006; Basak and Sathyanarayana 2010). At the extreme, more than half of antibiotic purchases were for <2 days (Greenhalgh 1987; Thamlikitkul 1988; Chuc and Tomson 1999). An interesting finding from Vietnam was that a higher percentage of incomplete antibiotic courses were sold for under 1s (Chuc and Tomson 1999).
6. Advice givingClients purchasing medicines in the pharmacy were found to receive very little counselling and advice from pharmacy staff. Provision of any unsolicited advice ranged widely from 2.5% to over 70%. Where it was given, however, it was usually limited to dose and frequency instructions; very few staff mentioned potential side effects, drug or food interactions, or advised to visit a physician if symptoms continued, worsened or complications arose (Dua *et al.* 1994; Modal and Lamba 1994; Kafle *et al.* 1996; Ross-Degnan *et al.* 1996; Duong *et al.* 1997a; Wachter *et al.* 1999; Chalker *et al.* 2000; Chuc *et al.* 2001; Ratanajamit and Chongsuvivatwong 2001; Van Sickle 2006; Basak and Sathyanarayana 2010; Hussain and Ibrahim 2011; Puspitasari *et al.* 2011; Saengcharoen and Lerkiatbundit 2013; Huda *et al.* 2014). Disease and medicine-specific advice and counselling was also poor, especially for STIs and contraceptive requests (Tuladhar *et al.* 1998; Chalker *et al.* 2000; Rahman *et al.* 2000; Ratanajamit and Chongsuvivatwong 2001; Minh *et al.* 2013). Advice regarding dietary and fluid intake for clients suffering from diarrhoea or anaemia was given by a minority of pharmacy staff (Kafle *et al.* 1996; Duong *et al.* 1997a; Wachter *et al.* 1999; Saengcharoen and Lerkiatbundit 2010; Minh *et al.* 2013). Finally, there was little evidence of signposting to other health services. This is highlighted by a study from Vietnam where simulated clients requested an abortifacient. None of the 30 pharmacies visited offered referral materials or contact details for a specialist service (Ngo *et al.* 2012).

### Part two: determinants of poor pharmacy performance in LMIC in Asia

#### Overview of included studies

The literature search yielded 38 relevant papers, from 28 distinct studies which conducted research in 11 countries: Pakistan (two), Thailand (five), Nepal (four), India (seven), Bangladesh (three), Lao PDR (one), Sri Lanka (one), Yemen Arab Republic (one), Vietnam (six), The Philippines (one) and Indonesia (one). The studies included in this review are very varied in terms of methodology and approach but they all shed some light on the determinants of pharmacy practice in these settings. Ten studies collected data on both ‘actual’ pharmacy practice (e.g. using mystery shopper surveys or spending time observing transactions) and knowledge and stated practice (through semi-structured interviews with store staff), with discordance between the two providing insight into factors affecting certain poor practices. In uncontrolled analyses, three studies tested for associations between a number of predictor variables and provider practices. Four studies conducted regression analyses using aspects of practice as the dependent variable and tested a number of explanatory variables (such as retailer characteristics or attitudes) as potential predictors of behaviour. Twelve studies evaluated the effectiveness of an intervention strategy and this provided evidence for whether or not these were important determinants of practice. Finally, 10 studies employed qualitative methodology, including in-depth interviews, participant and non-participant observation and focus group discussions.

From the literature, information on determinants of poor pharmacy practice can be distilled into three main categories: knowledge, profit motives and state intervention. The role of each is discussed.

1. KnowledgeOne possible explanation for the poor pharmacy practice observed in Asia is simply lack of knowledge. There is wealth of evidence, however, to suggest that knowing what constitutes good performance, whilst necessary, is not sufficient to ensure that this knowledge is employed in practice. A number of studies report vast discrepancies between knowledge or stated practice and actual practice; the qualifications of staff or level of training accomplished appears to make little difference to treatment behaviour and finally, educational programmes, whilst improving practice in the short-term, do not improve practice to a satisfactory level in the long-term. Each of these bodies of evidence will be examined in turn.
  - Discrepancies between knowledge and practiceSeveral studies employed different methods in order to elicit information on both provider knowledge or stated practice and actual practice. Vast discrepancies were noted between the two, suggesting that knowledge is not the key determinant of poor practice. Differences between stated practice and actual practice were observed for the sale of medicines, referral for medical advice, history taking and advice giving. For example, in hypothetical scenarios, 32% of pharmacists said they would sell any drugs for a man with urethral discharge and pain on urinating (the recommended management would be referral to a physician) (Chalker *et al.* 2000), 20% antibiotics for a child with a viral upper respiratory tract infection (Chuc *et al.* 2001), 40% corticosteroids and 0% antiepileptic medicines without a prescription (Larsson *et al.* 2006; Mac *et al.* 2006). In mystery shopper surveys, however, these actions were carried out by 85%, 83%, 98% and 21%, respectively. Adherence to recommended treatment practices was also found to be poorer in practice. Compared to the stated medication treatments for childhood diarrhoea, fewer pharmacy staff sold oral rehydration salts (ORS) and more sold inappropriate antibiotics and anti-diarrhoels (Ross-Degnan *et al.* 1996; Saengcharoen and Lerkiatbundit 2010; Minh *et al.* 2013). Similar patterns were observed in the management of other conditions, such as STIs (Khan *et al.* 2006). One study reported that despite 81% of pharmacists knowing that antibiotics were not effective in short courses, 48% of courses dispensed were for <5 days. Stated referral practices of patients presenting with conditions that need to be treated by a doctor were three to four times higher than in reality (Chalker *et al.* 2000; Khan *et al.* 2006). Finally, history taking and advice giving in questionnaires was found to be superior to the service simulated clients experienced (Ratanajamit and Chongsuvivatwong 2001; Khan *et al.* 2006).
  - Qualifications/training and experienceStudies from Thailand and Nepal, using simulated shoppers to investigate the management of childhood diarrhoea, pregnancy-related anaemia and requests for contraceptives, revealed that staff with higher levels of training or qualifications asked more questions and gave better advice but little differences were observed in terms of appropriate medication dispensing (Kafle *et al.* 1996; Ratanajamit and Chongsuvivatwong 2001; Saengcharoen and Lerkiatbundit 2010). Other studies found no association between qualifications of pharmacy personnel and the quality of history taking and counselling (Hussain and Ibrahim 2011; Saengcharoen and Lerkiatbundit 2013). Despite, recording no differences in actual practice, one of these papers reported that, in interviews designed to measure their knowledge, pharmacists achieved significantly higher scores compared to non-pharmacists in the areas of history taking and advice provision (Saengcharoen and Lerkiatbundit 2013). Experience was shown not to be a predictor of appropriate dispensing in the two studies which collected data on this variable (Apisarnthanarak *et al.* 2008; Saengcharoen and Lerkiatbundit 2010).
  - Impact of education programmesNine studies report the findings of educational interventions. All of these studies employed simulated client methodology to assess the impact of training; only four assessed performance by comparing outcomes to a control group. On the whole, training was found to improve the treatment behaviour of various conditions, including diarrhoea and STIs, as well as provision of contraceptives (Ross-Degnan *et al.* 1996; Kafle 1998; Tuladhar *et al.* 1998; Ratanajamit *et al.* 2002; Qidwai *et al.* 2006; Kafle *et al.* 2013; Minh *et al.* 2013; Pham *et al.* 2013). Despite the improvements noted, inadequacies in treatment practice remained. For example, in Indonesia, 46% of staff continued to sell anti-diarrhoels for children with diarrhoea (Ross-Degnan *et al.* 1996); and in Nepal, 55% of drug sellers continued to prescribe an STI treatment regimen that was inconsistent with national guidelines (Tuladhar *et al.* 1998). Further, most of the study follow-up times were <6 months. One study with follow-up at 7–9 months noted the waning of effect (Tuladhar *et al.* 1998), and another at 32 months reported no sustained improvements in the use of ORS, anti-diarrhoels or antibiotics for the treatment of diarrhoea, despite promising results at 6 months post intervention (Minh *et al.* 2013; Pham *et al.* 2013). In Nepal, small group training led to significant improvements in a number of aspects of management of childhood diarrhoea, acute respiratory infection and anaemia in pregnancy at 2 months but most of these effects were not sustained at the 5-month follow-up (Kafle 1998). One study did not report baseline data but the post-training results concerning the management of STIs were very poor (Khan *et al.* 2006). Another showed no significant impact of training on ORS use for the treatment of diarrhoea or dysentery, but it did show improvements in antibiotic dispensing, only for drug sellers, however, not for pharmacists (Podhipak *et al.* 1993).
2. Profit motivesPharmacies are retail businesses operating within a competitive marketplace. Several papers described the proliferation of medicine outlets over recent decades, especially in countries that underwent economic liberalisation. For example, in Vietnam following the privatization of drug provision in 1986, the number of private pharmacies increased from none to >6000 by 1996 (Chuc *et al.* 2002). These papers also noted the intensified competition that resulted (Kamat and Nichter 1998; Chuc and Tomson 1999; Stenson *et al.* 2001b; Chuc *et al.* 2002). An illustration of the nature of competition comes from Mumbai, India, where pharmacies hire agents to persuade patients leaving hospitals to patronise their pharmacy (Kamat and Nichter 1998). Qualitative work confirms that pharmacies report feeling intense competition and staff seek to maximize profit in order to survive in the market (Dua *et al.* 1994; Kamat and Nichter 1998; Chuc and Tomson 1999; Kotwani *et al.* 2012; Seeberg 2012). Essentially there are three ways to maximize profits: maximizing the number of customers, maximizing the revenue from each individual customer and minimizing costs. From the literature, we identified a number of strategies employed by pharmacy staff to achieve these goals, each of which can also be linked to poor practice.
  - Complying with customer demandsThe literature reveals that pharmacy staff are very responsive to patients wishes, adhering to a ‘customer is king’ mentality. In the name of maintaining clients, inducing loyalty and preventing customers from fulfilling their requests elsewhere, pharmacies resort to a number of poor practices. These include honouring improper prescriptions, such as those that are out of date, and selling POMs without a prescription (Dua *et al.* 1994; Larsson *et al.* 2006; Kotwani *et al.* 2012; Nga *et al.* 2014). Further, incomplete courses of antibiotics are frequently sold. Patients request these short courses due to economic constraints, a desire to test the therapeutic efficacy and presence of side-effects before purchasing larger quantities, and a belief that a full course is unnecessary (Lansang *et al.* 1990; Dua *et al.* 1994; Dineshkumar *et al.* 1995; Roy 1997;Duong *et al.* 1997b; Kamat and Nichter 1998; Mamun *et al.* 2006).
  - Selling medicines based on perceived efficacySeveral studies reported that pharmacy staff chose medicines based on their ability to produce a rapid recovery or temporary relief from symptoms, even where they were not appropriate (Kafle *et al.* 1996; Van Sickle 2006; Saengcharoen and Lerkiatbundit 2010,). For example, anti-diarrhoels for the treatment of childhood diarrhoea (Saengcharoen and Lerkiatbundit 2010) or airway relaxers for the respiratory symptoms associated with asthma (Van Sickle 2006). In addition, they sold medicines in which clients were believed to have great confidence, again, even if such medicines were not necessary. Examples include tonics as an accompaniment to antibiotics, and complex vitamin preparations (Dua *et al.* 1994; Kafle *et al.* 1996).
  - Mimicking doctorsFour studies reported that it was common practice for medicine retailers to study the prescriptions bought in by patients and then model their own prescribing on the practices of local doctors (Greenhalgh 1987; Kafle *et al.* 1996; Ross-Degnan *et al.* 1996; Seeberg 2012). This may simply be a way to improve knowledge. It could, however, be inferred that it is a strategy used by pharmacies in order to be viewed as more legitimate by customers.
  - Maintaining good relationships with doctorsWhen presented with clinically inappropriate prescriptions, pharmacies in Asia tended to dispense them rather than query their appropriateness with the doctor. Pharmacists interviewed in Kotwani et al’s qualitative study of irrational antibiotic use in Delhi described their low status in the medical hierarchy and how doctors would rebuke them for challenging their authority (Kotwani *et al.* 2012). Other research, also in India, has identified symbiotic relationships between doctors and chemist shops, and doctors have been observed to mention names of shops where patients should fill their prescriptions (Kamat and Nichter 1998; Seeberg 2012). Further, at the request of medical representatives, doctors reportedly prescribe more of particular products when local pharmacies experience an overstock (Kamat and Nichter 1998). It is understandable, in such a context, that pharmacists do not query more prescriptions for fear of aggravating local physicians.
  - Medicine salesTwo explicit strategies for maximizing profits from medicine sales were identified from the literature; selling large volumes of low priced drugs and recommending medicines that yield the greatest profit (Ross-Degnan *et al.* 1996; Chuc and Tomson 1999; Saengcharoen and Lerkiatbundit 2010). Antibiotics are singled out as high profit generators (Chuc and Tomson 1999; Chuc *et al.* 2001; Saengcharoen and Lerkiatbundit 2010; Nga *et al.* 2014); this may partly explain their rampant overuse.
  - Medicine purchasingThe pharmaceutical industry employs aggressive marketing techniques which involve promotional offers to pharmacies. This includes bonus schemes whereby the purchase of *x* amount of a product includes *y* amount for free (Kamat and Nichter 1998). Retailers are then incentivized to sell more of this product, regardless of its appropriateness, because it will yield high profits. The following quote from Kamat and Nichter’s (1998) ethnographic study of pharmacies and pharmaceutical-related behaviour in India gives an insight into such practice (the product mentioned, Superaction, an OTC product for cough, cold, fever and pain, is sold on a buy 12 strips get 7 free basis):I make a profit of anything between 75% and 100% on “Superaction.” During the past 2 week, I sold two boxes (20 strips) of this item for which I got a pocket calculator worth 80 rupees from the company. I make a lot of profit on this product, but I have to counter-push it because local doctors do not prescribe it. I do not recommend this product to every customer who asks for medicines for headache or body pain but mostly to angutachapwallas (illiterates) who come and ask me to give some medicine for cold and pain (Kamat and Nichter 1998).Dineshkumar *et al.* (1995) comment on the aggressive marketing of vitamins which are used extensively in India. Seeberg describes how chemists in Orissa purchase substandard medicines from local production facilities at a 50% discount and then sell them on to customers thus making additional profit (Seeberg 2012).It is important to note that there are examples in the literature which illustrate that medicine retailers are only prepared to go so far in risking the health of their patients in the name of making a quick profit. Pharmacists described how it was not suitable to use substandard medicines for patients who had undergone surgery or faced life-threatening conditions (Seeberg 2012). Additionally, when patients sought pharmacists’ advice in the event of not being able to afford all medicines on a prescription, they were found to recommend the medicines which ‘cure’ over those with the highest profit (Kamat and Nichter 1998).
3. State interventionA few studies in this review provide information on the impact of government intervention on pharmacy performance.
  - RegulationTwo intervention studies reported on the effect of regulation on service quality. An intervention in Lao PDR involving inspection visits, punishments in the case of gross violations of the sanctions, up-to-date supply of regulatory documents, and reinforcement of the rules found marked improvements in the availability of essential medicines, order in the pharmacy and provision of information; and less mixing of different drugs in the same package (Stenson *et al.* 2001b). The regulatory component of a multi-component (sequentially applied), intervention on dispensing practices in Bangkok (Thailand) was the only component of the intervention that resulted in a significant change in practice (reduced dispensing of a prescription-only steroid compared to the control group). This intervention focussed on the illegality of the act and the threat of punishment should such practice be observed. The same study reported that in Hanoi (Vietnam), where less focus was placed on sanctions, the regulatory component of the intervention did not lead to an immediate change in behaviour (Chalker *et al.* 2005).
  - National Public Health ProgrammesA study from Vietnam investigating the management of tuberculosis patients in the pharmacy found, in a multivariate analysis, that staff who were aware both of the National Tuberculosis Programme (NTP) and that tuberculosis medicines were provided for free were 5.8 times more likely to refer a suspect directly to a tuberculosis facility than those who were not (Vu *et al.* 2012). Another study concerned with management of childhood diarrhoea investigated practice in three countries: Bangladesh, Sri Lanka and Yemen Arab Republic. The authors reported that ORS was more commonly dispensed in Bangladesh and they noted the presence of a national ORS programme as one potential explanation (Tomson and Sterky 1986).

## Discussion

Combined, the reviews identified 60 papers reporting on pharmacy practice and/or determinants of poor practice in 15 LMICs in Asia. The majority of studies were from lower middle-income countries. Asides from studies from Mongolia, Yemen Arab Republic and Syria, all other studies focussed on countries from South and South-East Asia. As such, the results tell us little about pharmacy practice in North Asia, Central Asia, West Asia or the Middle East. Most research was carried out on PRPs, with less on NPRPs and research on unregistered shops was found to be practically non-existent.

Given the diversity of studies found in the search, quality appraisal proved to be a particular challenge. Relevant papers included a range of designs including randomised controlled trials, cross-sectional descriptive surveys and ethnographies. The lack of clear criteria by which qualitative studies should be judged in the systematic review process has been raised by others but remains an unresolved issue (Dixon-Woods *et al.* 2006). In light of this, it was decided to include all papers which met the inclusion criteria providing the methodology employed was clear. Data on both methods and study design were extracted and any potential threats to validity were recorded. The main concerns noted were small sample sizes and non-random sampling (quantitative papers). The findings of poorer quality studies, however, were found to be consistent with more rigorously designed ones. In interpreting the findings, care has been taken to emphasise those which were found in a number of studies and across countries.

In terms of pharmacy performance, the findings across countries and over time are remarkably consistent. Pharmacy practice in Asia appears to have changed little in the past 30 years. The same problems documented by studies in the 1980s are true of practice in recent times (Tomson and Sterky 1986; Greenhalgh 1987; Hussain and Ibrahim 2012; Seeberg 2012; Vu *et al.* 2012; Chua *et al.* 2013; Minh *et al.* 2013; Saengcharoen and Lerkiatbundit 2013). Practice appears to fall short throughout the pharmacy encounter. The key inadequacies documented throughout the literature are: insufficient history taking prior to the sale of medicines; a lack of referral of patients whose management is outside of the remit of a pharmacist’s expertise; the illegal sale of a wide range of POMs without a prescription; the sale of medicines that are either clinically inappropriate and/or in doses that are outside of the therapeutic range; the sale of incomplete courses of antibiotics; and finally, limited provision of information and counselling to accompany the sale of medicines. Similar challenges have also been documented in Sub-Saharan Africa (Wafula *et al.* 2012)

Staff working in pharmacies can be seen as the gatekeepers of medicines. They stand at the interface between producers and consumers of medicines and their role is to ensure that they are used safely, effectively and rationally (Anderson 2002). When used correctly, medicines can save lives and improve people’s health; irrational use, however, can have harmful consequences. A number of conditions were found to be treated inadequately in the pharmacy, including diarrhoea, asthma, anaemia, tuberculosis, STIs, RTIs and migraine. This mistreatment can lead to unnecessary morbidity and mortality. For example, many studies reporting on the management of childhood diarrhoea found under-provision of ORS and over-provision of anti-diarrhoeals and antibiotics. The use of anti-diarrhoels in infants has been shown to be harmful (Minton and Smith 1987; Li *et al.* 2007) and it is estimated that correct treatment with ORS may prevent 93% of diarrhoeal deaths in children under 5 (Munos *et al.* 2010). In South Asia, diarrhoea is thought to account for 23% of all deaths in children under 5 (Morris *et al.* 2003).

Overuse of antibiotics is a particular concern for public health, as misuse of antibiotics over recent decades has led to the selection and spread of resistant bacteria. As a result, antibacterial drugs have become less effective and in some cases, ineffective. Earlier this year, a WHO global report on the surveillance of antibiotic resistance described the problem as a ‘global health security emergency’ (World Health Organization 2014).

A further concern is the economic impact of spending on households, especially the poor. Customers typically pay for the medicines purchased at pharmacies out of their own pocket and where these medicines are inappropriate or ineffective this represents a waste of scarce resources. Work in Asia has shown that, in many countries, a large proportion of out of pocket payments is spent on medicines. For example, in Bangladesh, Vietnam and India, this share is 70% (Van Doorslaer *et al.* 2007). Further, in these countries, out of pocket payments for healthcare can be ‘catastrophic’, accounting for >25% of household resources (excluding food costs) in at least 10% of all households (ibid).

Turning to the determinants of the poor practice documented above, the picture is less clear. Despite the importance of pharmacies and the potential benefit for public health if practice were to improve, efforts to understand and address the problem have been surprisingly few. Historically, the small number of attempts to improve pharmacy practice in Asia has focussed on training interventions. This review finds that whilst a necessary condition, adequate knowledge alone is not sufficient to ensure appropriate management of patients presenting at the pharmacy. Profit motives and the regulatory environment appear to play a role but the research evidence is relatively sparse. In terms of the methods used to unpick the underlying determinants of pharmacy behaviours, we found that in-depth qualitative studies, particularly those employing an ethnographic approach, provided the richest data (Kamat and Nichter 1998; Seeberg 2012). Unfortunately, studies using this approach are rare.

Whilst a number of studies have been published in the two decades since Goel *et al.* (1996) first reviewed this literature, there is little new insight into the problem of poor pharmacy practice. They noted that regulatory factors had been ‘strikingly neglected by researchers’ and called for new research on the ‘impact of professional ownership on professional freedom’. Researchers have, on the whole, continued to neglect these areas. The pursuit of regulatory enforcement is, however, not a straightforward solution and we must be aware that enforcing laws surrounding the sale of POMs would potentially deny many people access to essential medicines, thus violating a basic principle of public health.

Based on the intervention literature both within and outside Asia, the menu of evidence-based options for professional bodies and policy-makers to inform improvement and development of pharmacy services is limited (Smith 2009a). Arguably, some cadre of trained pharmacy workers should be in place in order to provide a basis for improvement, yet in many settings human resource limitations undermine the ability to provide this (Smith 2009b). Asides from training and regulation, other schemes that have been implemented include peer review, accreditation (such as the Accredited Drug Dispensing Outlets scheme in Tanzania) and social franchising (Chalker *et al.* 2005; Wafula and Goodman 2010). However, the evidence on the impact and sustainability of these strategies remains quite limited, highlighting this area as an important priority for future research (Center for Pharmaceutical Management 2008; Wafula and Goodman 2010; Valimb *et al.* 2014).

It is worth noting that new organisational arrangements of pharmacy retail in the form of chains and franchises are a growing phenomenon in LMIC both in Asia and elsewhere (Lowe and Montagu 2009; IMS Consulting Group 2014). This phenomenon raises important questions about the impact of professional and organised ownership on pharmacy practice. Further, theoretical literature suggests that the organisational structure of the pharmacy firm may affect both the regulatory environment and financial incentives, as well as provider knowledge (Klein 1980; Blair and Kaserman 1994; Frant 1996; Bloom *et al.* 2008).

Cross and Macgregor (2010) have criticised the current debates around ‘informal providers’ (including drug sellers) which, they argue, are myopically focused on ‘small time economic actors’ rather than giving attention higher up in the pharmaceutical supply chain (Cross and Macgregor 2010). This focus indeed leads to a distraction away from the pharmaceutical industry, which, thus far, has largely remained absent from discussions of inappropriate medicines use. A few papers in this review touch on the pressures that providers face from industry but this does not come out strongly. Whilst it is necessary to study frontline behaviours, research upstream is also a necessity.

## Conclusion

Pharmacies are an important component of the health system in LMIC in Asia. In many areas they act as ‘*de facto* primary healthcare providers’ (Seeberg *et al.* 2014). The service they provide, however, does not live up to international expectations of pharmacy practice. The consequences of poor practice can have harmful effects for public health and, as such, these outlets warrant more attention from public health researchers. The nature of the problem with pharmacies in Asia is well established, although more attention could be paid to NPRPs pharmacies and unregistered drug shops. Future research efforts should focus their attention on investigating the underlying causes and ways to improve the current situation. The little evidence that is available suggests that intervention strategies should take into account the regulatory environment and profit incentives faced by pharmacy personnel and not continue to focus solely on improving knowledge. If efforts are focussed accordingly it is hoped that the realities of the past 30 years of poor pharmacy practice in Asia will not continue for the next 30.

## Supplementary data

Supplementary data are available at *HEAPOL* online.

Supplementary Data

## References

[czw007-B1] Al-Faham Z Habboub G Takriti F. 2011 The sale of antibiotics without prescription in pharmacies in Damascus, Syria. Journal of Infection in Developing Countries 5: 396–9. 21628818 10.3855/jidc.1248

[czw007-B2] Anderson S. 2002 The state of the world's pharmacy: a portrait of the pharmacy profession. Journal of Interprofessional Care 16: 391–404. 12487847 10.1080/1356182021000008337

[czw007-B3] Apisarnthanarak A Tunpornchai J Tanawitt K Mundy LM. 2008 Nonjudicious dispensing of antibiotics by drug stores in Pratumthani, Thailand. Infection Control and Hospital Epidemiology 29: 572–5. 18510468 10.1086/587496

[czw007-B4] Basak SC Sathyanarayana D. 2010 Evaluating medicines dispensing patterns at private community pharmacies in Tamil Nadu, India. Southern Medicine Review 3: 27–31.

[czw007-B5] Bigdeli M Peters D Wagner A. 2014 Medicines in Health Systems: Advancing access, affordability and appropriate use. Geneva: World Health Organization.

[czw007-B6] Bista KP Chaudhary P Slanger TE Khan MH. 2002 The practice of STI treatment among chemists and druggists in Pokhara, Nepal. Sex Transmitted Infections 78: 223 10.1136/sti.78.3.223 PMC1744481 12238660

[czw007-B7] Blair RD Kaserman DL. 1994 A Note on Incentive Incompatibility under Franchising. Review of Industrial Organization 9: 323–30.

[czw007-B8] Bloom G Standing H Lloyd R. 2008 Markets, information asymmetry and health care: towards new social contracts. Social Science and Medicine 66: 2076–87. 18316147 10.1016/j.socscimed.2008.01.034

[czw007-B9] Center for Pharmaceutical Management. 2008 Accredited Drug Dispensing Outlets in Tanzania Strategies for Enhancing access to Medicines Program Prepared for the Strategies for Strategies for Enhancing Access to Medicines Program. Arlington, VA: Management Sciences for Health[TQ1]

[czw007-B10] Chalker J Chuc N Falkenberg T Do N Tomson G. 2000 STD management by private pharmacies in Hanoi: practice and knowledge of drug sellers. Sexually Transmitted Infections 76: 299–302. 11026888 10.1136/sti.76.4.299 PMC1744190

[czw007-B11] Chalker J Ratanawijitrasin S Chuc N Petzold M Tomson G. 2005 Effectiveness of a multi-component intervention on dispensing practices at private pharmacies in Vietnam and Thailand—a randomized controlled trial. Social Science and Medicine 60: 131–41. 15482873 10.1016/j.socscimed.2004.04.019

[czw007-B12] Chua SS Lim KP Lee HG 2013 Utilisation of community pharmacists by the general public in Malaysia. The International Journal of Pharmacy Practice 21:66–9. 23301536 10.1111/j.2042-7174.2012.00219.x

[czw007-B13] Chuc N Larsson M Falkenberg T 2001 Management of childhood acute respiratory infections at private pharmacies in Vietnam. Annals of Pharmacotherapy 35: 1283–8. 11675861 10.1345/aph.10313

[czw007-B14] Chuc NT Larsson M Do NT 2002 Improving private pharmacy practice: a multi-intervention experiment in Hanoi, Vietnam. Journal of Clinical Epidemiology 55: 1148–55. 12507680 10.1016/s0895-4356(02)00458-4

[czw007-B15] Chuc NTK Tomson G. 1999 “Doi moi” and private pharmacies: a case study on dispensing and financial issues in Hanoi, Vietnam. European Journal of Clinical Pharmacology 55: 325–32. 10424327 10.1007/s002280050636

[czw007-B16] Cockburn R Newton PN Agyarko EK Akunyili D White NJ. 2005 The global threat of counterfeit drugs: why industry and governments must communicate the dangers. PLoS Medicine 2: e100 15755195 10.1371/journal.pmed.0020100 PMC1062889

[czw007-B17] Cong LD Yen PT Nhu TV Binh LN. 1998 Use and quality of antimalarial drugs in the private sector in Viet Nam. Bull World Health Organisation 76: 51–8. PMC2305575 9763723

[czw007-B18] Cross J Macgregor HN. 2010 Knowledge, legitimacy and economic practice in informal markets for medicine: a critical review of research. Social Science and Medicine 71: 1593–600. 20855143 10.1016/j.socscimed.2010.07.040

[czw007-B19] Dineshkumar B Raghuram TC Radhaiah G Krishnaswamy K. 1995 Profile of drug use in urban and rural India. Pharmacoeconomics 7: 332–46. 10155322 10.2165/00019053-199507040-00007

[czw007-B20] Dixon-Woods M Bonas S Booth A 2006 How can systematic reviews incorporate qualitative research? A critical perspective. Qualitative Research 6: 27–44.

[czw007-B21] Dua V Kunin CM White LV. 1994 The use of antimicrobial drugs in Nagpur, India. A window on medical care in a developing country. Social Science and Medicine 38: 717–24. 8171350 10.1016/0277-9536(94)90462-6

[czw007-B22] Duong D Le T Binns CW. 1997a Diarrhoea management by pharmacy staff in retail pharmacies in Hanoi, Vietnam. International Journal of Pharmacy Practice 5: 97–100.

[czw007-B23] Duong DV Binns CW Le TV. 1997b Availability of antibiotics as over‐the‐counter drugs in pharmacies: a threat to public health in Vietnam. Tropical Medicine and International Health 2: 1133–9. 9438468 10.1046/j.1365-3156.1997.d01-213.x

[czw007-B24] Ferguson AE. 1981 Commercial pharmaceutical medicine and medicalization: a case study from El Salvador. Culture, Medicine and Psychiatry 5: 105–34. 10.1007/BF00055416 7261658

[czw007-B25] Frant H. 1996 High-powered and low-powered incentives in the public sector. Journal of Public Administration Research and Theory 6: 365–81.

[czw007-B26] Goel P Ross-Degnan D Berman P Soumerai S. 1996 Retail pharmacies in developing countries: a behavior and intervention framework. Social Science and Medicine 42: 1155–61. 8737433 10.1016/0277-9536(95)00388-6

[czw007-B27] Greenhalgh T. 1987 Drug prescription and self-medication in India: an exploratory survey. Social Science and Medicine 25: 307–18. 3629304 10.1016/0277-9536(87)90233-4

[czw007-B28] Haak H. 1988 Pharmaceuticals in two Brazilian villages: lay practices and perceptions. Social Science and Medicine 27: 1415–27. 3238460 10.1016/0277-9536(88)90208-0

[czw007-B29] Hadi U Broek PVD Kolopaking EP 2010 Cross-sectional study of availability and pharmaceutical quality of antibiotics requested with or without prescription (over the counter) in Surabaya, Indonesia. BMC Infectious Diseases 10:203. 20618975 10.1186/1471-2334-10-203 PMC2914770

[czw007-B30] Holloway K Van Dijk L. 2011 The world medicines situation 2011 Rational Use of Medicines. Geneva: WHO.

[czw007-B31] Huda FA Ngo TD Anisuddin A Anadil A Reichenbach AL 2014 Availability and provision of misoprostol and other medicines for menstrual regulation among pharmacies in Bangladesh via mystery client survey. International Journal of Gynecology and Obstetrics 124: 164–8. 24268354 10.1016/j.ijgo.2013.07.037

[czw007-B32] Hussain A Ibrahim MI. 2011 Medication counselling and dispensing practices at community pharmacies: a comparative cross sectional study from Pakistan. International Journal of Clinical Pharmacy 33: 859–67. 21853362 10.1007/s11096-011-9554-6

[czw007-B33] Hussain A Ibrahim MI. 2012 Management of diarrhoea cases by community pharmacies in 3 cities of Pakistan. Eastern Mediterranean Health Journal 18: 635–40. 22888622 10.26719/2012.18.6.635

[czw007-B34] Igun U. 1987 Why we seek treatment here: retail pharmacy and clinical practice in Maiduguri, Nigeria. Social Science and Medicine 24: 689–95. 3603091 10.1016/0277-9536(87)90312-1

[czw007-B35] IMS Consulting Group 2014. Assessing the growth of pharmacy chains New York: IMS Consulting Group.

[czw007-B36] Institute of Medicines 2013 Countering the Problem of Falsified and Substandard Drugs. Washington DC: The National Academies Press. 24872973

[czw007-B37] Kafle K. 1998 *Impact of Action-Orientated Training and/or Mailed Print Material on Retailer Practices: Safe Dispensing, Correct Advice, and Appropriate Referral for Diarrhoea, ARI and Pregnancy*. Kathmandu: INRUD.

[czw007-B38] Kafle K Karkee S Shrestha N 2013 Improving private drug sellers’ practices for managing common health problems in Nepal. Journal of Nepal Health Research Council 11: 198–204. 24362611

[czw007-B39] Kafle KK Madden JM Shrestha AD 1996 Can licensed drug sellers contribute to safe motherhood? A survey of the treatment of pregnancy-related anaemia in Nepal. Social Science and Medicine 42: 1577–88. 8771641 10.1016/0277-9536(95)00294-4

[czw007-B40] Kamat VR Nichter M. 1998 Pharmacies, self-medication and pharmaceutical marketing in Bombay, India. Social Science and Medicine 47: 779–94. 9690824 10.1016/s0277-9536(98)00134-8

[czw007-B41] Khan MM Grubner O Kramer A. 2012 Frequently used healthcare services in urban slums of Dhaka and adjacent rural areas and their determinants. Journal of Public Health 34: 261–71. 22241915 10.1093/pubmed/fdr108

[czw007-B42] Khan MM Wolter S Mori M. 2006 Post-training quality of syndromic management of sexually transmitted infections by chemists and druggists in Pokhara, Nepal: is it satisfactory? International Journal of Quality in Health Care 18: 66–72. 10.1093/intqhc/mzi086 16254006

[czw007-B43] Klein B. 1980 Transaction cost determinants of” unfair” contractual arrangements. The American Economic Review 70: 356–62.

[czw007-B44] Kloos H Chama T Abemo D Tsadik KG Belay S. 1986 Utilization of pharmacies and pharmaceutical drugs in Addis Ababa, Ethiopia. Social Science and Medicine 22: 653–72. 3715505 10.1016/0277-9536(86)90038-9

[czw007-B45] Kotwani A Wattal C Joshi PC Holloway K. 2012 Irrational use of antibiotics and role of the pharmacist: an insight from a qualitative study in New Delhi, India. Journal of Clinical Pharmacy and Therapeutics 37: 308–12. 21883328 10.1111/j.1365-2710.2011.01293.x

[czw007-B46] Krishnaswamy K Kumar BD Radhaiah G. 1985 A drug survey—precepts and practices. European Journal of Clinical Pharmacology 29: 363–70. 4076333 10.1007/BF00544095

[czw007-B47] Lansang MA Lucas-Aquino R Tupasi TE 1990 Purchase of antibiotics without prescription in Manila, the Philippines. Inappropriate choices and doses. Journal of Clinical Epidemiology 43: 61–7. 2319282 10.1016/0895-4356(90)90057-v

[czw007-B48] Larsson M Tomson G Binh N Chuc N Falkenberg T. 2006 Private pharmacy staff in Hanoi dispensing steroids-theory and practice. Pharmacy Practice 4: 60–7. 25247001 PMC4166145

[czw007-B49] Li STT Grossman DC Cummings P. 2007 Loperamide therapy for acute diarrhea in children: systematic review and meta-analysis. PLoS Medicine 4: e98 17388664 10.1371/journal.pmed.0040098 PMC1831735

[czw007-B50] Logan K. 1983 Part 5: The role of pharmacists and over the counter medications in the health care system of a Mexican city. Medical Anthropology 7: 68–89.

[czw007-B51] Lowe RF Montagu D. 2009 Legislation, regulation, and consolidation in the retail pharmacy sector in low-income countries. Southern Med Review 2: 35–44.

[czw007-B52] Mac TL Le VT Vu AN Preux PM Ratsimbazafy V. 2006 AEDs Availability and Professional Practices in Delivery Outlets in a City Center in Southern Vietnam. Epilepsia 47: 330–4. 16499757 10.1111/j.1528-1167.2006.00425.x

[czw007-B53] Mamun KZ Tabassum S Shears P Hart CA. 2006 A survey of antimicrobial prescribing and dispensing practices in rural Bangladesh. Mymensingh Medical Journal 15: 81–4. 16467769 10.3329/mmj.v15i1.9

[czw007-B54] Mayhew S Nzambi K Pepin J Adjei S. 2001 Pharmacists’ role in managing sexually transmitted infections: policy issues and options for Ghana. Health Policy and Planning 16: 152–60. 11358916 10.1093/heapol/16.2.152

[czw007-B55] Minh PD Huong DTM Byrkit R Murray M. 2013 Strengthening pharmacy practice in Vietnam: findings of a training intervention study. Tropical Medicine and International Health 18: 426–34. 23294473 10.1111/tmi.12062

[czw007-B56] Minton N Smith P. 1987 Loperamide toxicity in a child after a single dose. British Medical Journal (Clinical Research Ed.) 294: 1383 3109665 10.1136/bmj.294.6584.1383 PMC1246550

[czw007-B57] Modal T Lamba L. 1994 Practices of chemist regarding diarrhoea management. Indian Pediatrics 31: 1535–6. 7875814

[czw007-B58] Morris SS Black RE Tomaskovic L. 2003 Predicting the distribution of under-five deaths by cause in countries without adequate vital registration systems. International Journal of Epidemiology 32: 1041–51. 14681271 10.1093/ije/dyg241

[czw007-B59] Munos MK Walker CLF Black RE. 2010 The effect of oral rehydration solution and recommended home fluids on diarrhoea mortality. International Journal of Epidemiology 39: i75–87. 20348131 10.1093/ije/dyq025 PMC2845864

[czw007-B60] Nakajima R Takano T Urnaa V Khaliun N Nakamura K. 2010 Antimicrobial use in a country with insufficient enforcement of pharmaceutical regulations: a survey of consumption and retail sales in Ulaanbaatar, Mongolia. Southern Med Review 3: 19–23. 23093879 PMC3471171

[czw007-B61] Newton PN Green MD Fernández FM Day NP White NJ. 2006 Counterfeit anti-infective drugs. The Lancet Infectious Diseases 6: 602–13. 16931411 10.1016/S1473-3099(06)70581-3

[czw007-B62] Nga D Chuc N Hoa N 2014 Antibiotic sales in rural and urban pharmacies in northern Vietnam: an observational study. BMC Pharmacology and Toxicology 15:10.1186/2050-6511-15-6 PMC3946644 24555709

[czw007-B63] Ngo TD Park MH Nguyen TH. 2012 Pharmacy workers' knowledge and provision of abortifacients in Ho Chi Minh City, Vietnam. International Journal of Gynaecology and Obsteterics 117: 187–8. 10.1016/j.ijgo.2011.12.017 22342054

[czw007-B64] Pham DM Byrkit M van Pham H Pham T Nguyen CT 2013 Improving pharmacy staff knowledge and practice on childhood diarrhea management in Vietnam: are educational interventions effective? PLoS One 8: e74882 24098355 10.1371/journal.pone.0074882 PMC3789740

[czw007-B65] Podhipak A Varavithya W Punyaratabandhu P Vathanophas K Sangchai R. 1993 Impact of an educational program on the treatment practices of diarrheal diseases among pharmacists and drugsellers. Southeast Asian Journal of Tropical Medicine Public Health 24: 32–9. 8362303

[czw007-B66] Price LJ. 1989 In the shadow of biomedicine: self medication in two Ecuadorian pharmacies. Social Science and Medicine 28: 905–15. 2711226 10.1016/0277-9536(89)90315-8

[czw007-B67] Puspitasari HP Faturrohmah A Hermansyah A. 2011 Do Indonesian community pharmacy workers respond to antibiotics requests appropriately? Tropical Medicine and International Health 16: 840–6. 21545380 10.1111/j.1365-3156.2011.02782.x

[czw007-B68] Qidwai W Krishanani M Hashmi S Afridi M Ali R. 2006 Private drug sellers' education in improving prescribing practices. Journal of the College of Physicians and Surgeons–Pakistan 16: 743–6. 17125630

[czw007-B69] Radyowijati A Haak H. 2003 Improving antibiotic use in low-income countries: an overview of evidence on determinants. Social Science and Medicine 57: 733–44. 12821020 10.1016/s0277-9536(02)00422-7

[czw007-B70] Rahman S Ahmed M Khuda B. 2000 Can medicine-sellers in pharmacies of urban Bangladesh meet the needs of clients with STD? World Health and Population 3: 1–6.

[czw007-B71] Ratanajamit C Chongsuvivatwong V. 2001 Survey of knowledge and practice on oral contraceptive and emergency contraceptive pills of drugstore personnel in Hat Yai, Thailand. Pharmacoepidemiology Drug Safety 10: 149–56. 11499854 10.1002/pds.573

[czw007-B72] Ratanajamit C Chongsuvivatwong V Geater AF. 2002 A randomized controlled educational intervention on emergency contraception among drugstore personnel in southern Thailand. Journal of the American Medical Womens Association 57: 196–9. 207. 12405236

[czw007-B73] Rathnakar UP Sharma NK Garg R Unnikrishnan B Gopalakrishna HN. 2012 A study on the sale of antimicrobial agents without prescriptions in pharmacies in an urban area in south India. Journal of Clinical and Diagnostic Research 6: 951–4.

[czw007-B74] Ross-Degnan D Soumerai SB Goel PK 1996 The impact of face-to-face educational outreach on diarrhoea treatment in pharmacies. Health Policy and Planning 11: 308–18. 10160376 10.1093/heapol/11.3.308

[czw007-B75] Roy J. 1997 Health status, treatment and drug use in rural Bangladesh: a case study of a village. Australian Journal of Rural Health 5: 70–5. 9444124 10.1111/j.1440-1584.1997.tb00241.x

[czw007-B76] Saengcharoen W Lerkiatbundit S. 2010 Practice and attitudes regarding the management of childhood diarrhoea among pharmacies in Thailand. International Journal of Pharmacy Practice 18: 323–31. 21054592 10.1111/j.2042-7174.2010.00066.x

[czw007-B77] Saengcharoen W Lerkiatbundit S. 2013 Migraine management in community pharmacies: practice patterns and knowledge of pharmacy personnel in Thailand. Headache 53: 1451–63. 23808927 10.1111/head.12163

[czw007-B78] Saradamma RD Higginbotham N Nichter M. 2000 Social factors influencing the acquisition of antibiotics without prescription in Kerala State, south India. Social Science and Medicine 50: 891–903. 10695985 10.1016/s0277-9536(99)00380-9

[czw007-B79] Seeberg J. 2012 Connecting pills and people: an ethnography of the pharmaceutical nexus in Odisha, India. Medical Anthropology Quarterly 26: 182–200. 22905436 10.1111/j.1548-1387.2012.01200.x

[czw007-B80] Seeberg J Pannarunothai S Padmawati RS 2014 Treatment seeking and health financing in selected poor urban neighbourhoods in India, Indonesia and Thailand. Social Science and Medicine 102: 49–57. 24565141 10.1016/j.socscimed.2013.11.039

[czw007-B81] Smith F. 2009a Private local pharmacies in low‐and middle‐income countries: a review of interventions to enhance their role in public health. Tropical Medicine and International Health 14: 362–72. 19207171 10.1111/j.1365-3156.2009.02232.x

[czw007-B82] Smith F. 2009b The quality of private pharmacy services in low and middle-income countries: a systematic review. Pharmacy World and Science 31: 351–61. 19343530 10.1007/s11096-009-9294-z

[czw007-B83] Sreeramareddy CT Shankar RP Sreekumaran BV 2006 Care seeking behaviour for childhood illness-a questionnaire survey in western Nepal. BMC International Health and Human Rights 6: 7 16719911 10.1186/1472-698X-6-7 PMC1543657

[czw007-B84] Stenson B Syhakhang L Eriksson B Tomson G. 2001a Real world pharmacy: assessing the quality of private pharmacy practice in the Lao People's Democratic Republic. Social Science and Medicine 52: 393–404. 11330774 10.1016/s0277-9536(00)00142-8

[czw007-B85] Stenson B Syhakhang L Lundborg CS Eriksson B Tomson G. 2001b Private pharmacy practice and regulation. A randomized trial in Lao P.D.R. International Journal of Technology Assessment in Health Care 17: 579–89. 11758301

[czw007-B86] Syhakhang L Stenson B Wahlström R Tomson G. 2001 The quality of public and private pharmacy practices. European Journal of Clinical Pharmacology 57: 221–7. 11497337 10.1007/s002280100295

[czw007-B87] Thamlikitkul V. 1988 Antibiotic dispensing by drug store personnel in Bangkok, Thailand. Journal of Antimicrobial Chemotherapy 21: 125–31. 3356619 10.1093/jac/21.1.125

[czw007-B88] Tomson G Sterky G. 1986 Self-prescribing by way of pharmacies in three Asian developing countries. The Lancet 328: 620–2. 10.1016/s0140-6736(86)92438-4 2875329

[czw007-B89] Tuladhar SM Mills S Acharya S 1998 The role of pharmacists in HIV/STD prevention: evaluation of an STD syndromic management intervention in Nepal. AIDS 12: S81–7. 9792365

[czw007-B90] Valimb AR Liana J Joshi M 2014 Engaging private sector drug dispensers to improve antimicrobial use in the community: Experience from the Piloted ADDO AMR Initiative in Tanzania. Journal of Pharmaceutical Policy and Practice 7: 11. 25298887 10.1186/2052-3211-7-11 PMC4177420

[czw007-B91] Van Der Geest S. 1982 Part 1: The illegal distribution of western medicines in developing countries: Pharmacists, drug pedlars, injection doctors and others. A bibliographic exploration. Medical Anthropology 6: 197–219.

[czw007-B92] Van Doorslaer E O'donnell O Rannan‐Eliya RP 2007 Catastrophic payments for health care in Asia. Health Economics 16: 1159–84. 17311356 10.1002/hec.1209

[czw007-B93] Van Sickle D. 2006 Management of asthma at private pharmacies in India. International Journal of Tuberculosis and Lung Disease 10: 1386–92. 17167957

[czw007-B94] Vu DH van Rein N Cobelens FG 2012 Suspected tuberculosis case detection and referral in private pharmacies in Viet Nam. International Journal of Tuberculosis and Lung Disease 16: 1625–9. 23131260 10.5588/ijtld.12.0295

[czw007-B95] Wachter DA Joshi MP Rimal B. 1999 Antibiotic dispensing by drug retailers in Kathmandu, Nepal. Tropical Medicine and International Health 4: 782–8. 10588773 10.1046/j.1365-3156.1999.00476.x

[czw007-B96] Wafula FN Goodman CA. 2010 Are interventions for improving the quality of services provided by specialized drug shops effective in sub-Saharan Africa? A systematic review of the literature. International Journal for Quality in Health Care 22: 316–23. 20430823 10.1093/intqhc/mzq022 PMC2908156

[czw007-B97] Wafula FN Miriti EM Goodman CA. 2012 Examining characteristics, knowledge and regulatory practices of specialized drug shops in Sub-Saharan Africa: a systematic review of the literature. BMC Health Services Research 12: 223 22838649 10.1186/1472-6963-12-223 PMC3520114

[czw007-B98] Wolffers I. 1987 Drug information and sale practices in some pharmacies of Colombo, Sri Lanka. Social Science and Medicine 25: 319–21. 3629305 10.1016/0277-9536(87)90234-6

[czw007-B99] World Bank. 2014 *Country and Lending Groups* [Online]. http://data.worldbank.org/about/country-and-lending-groups, accessed 24 June 2014.

[czw007-B100] World Health Organization. 2006 Combating counterfeit drugs: a concept paper for effective international cooperation. *Concept Paper, WHO, Rome, January*, 27.

[czw007-B101] World Health Organization 2014. Antimicrobial resistance: global report on surveillance 2014. Geneva: World Health Organization.

