# Supplementary material for: Performance of retail pharmacies in low- and middle-income Asian settings: a systematic review
Source: Health Policy Plan. 2016 Mar 8;31(7):940–53. doi: 10.1093/heapol/czw007 (PMC4977427; doi:10.1093/heapol/czw007)
Supplement: Supplementary Data [file supp_31_7_940__index.html]

Performance of retail pharmacies in low- and middle-income Asian settings: a systematic review — Supplementary Data 

# Performance of retail pharmacies in low- and middle-income Asian settings: a systematic review

## Supplementary Data

files

- Supplementary Data - docx file
