# Supplementary material for: Performance of retail pharmacies in low- and middle-income Asian settings: a systematic review
Source: Health Policy Plan. 2016 Mar 8;31(7):940–53. doi: 10.1093/heapol/czw007 (PMC4977427; doi:10.1093/heapol/czw007)
Supplement: Supplementary Data [file supp_czw007_Appendix_1.docx]

**Appendix 1: Key characteristics of papers included in the review**

| **Author & date, country** | **Number and type of shops** | **Study design** | **Data collection methods** | **Part of review** |
| --- | --- | --- | --- | --- |
| Al-Faham et al 2011, Syria | 200 private pharmacies | Cross-sectional study | *Simulated client presenting as the sibling of a woman with sinusitis. *Questionnaire survey to ascertain information on pharmacy and staff characteristics and knowledge and practice regarding menstrual regulation. | 1 |
| Apisarnthanarak et al 2008, Thailand | 280 1st class drug stores | Cross-sectional study | *Simulated clients presenting with various conditions (acute viral pharyngitis; influenza; acute viral sinusitis; acute gastroenteritis; non-infected skin abrasion; acute UTI). | 1&2 |
| Basak and Sathyanarayana 2010, India | 24 private pharmacies | Cross-sectional study | *4 hours of observation per pharmacy *Structured interviews with pharmacy staff regarding medicines. | 1 |
| Bista et al 2002*, Nepal | 37 private pharmacies | Cross-sectional study (unclear if baseline results for Khan 2006) | *Simulated client presenting with history of urethral or vaginal discharge.  *Review of registry data kept on all STI clients by pharmacies. | 1 |
| Chalker et al 2000*, Vietnam | 60 private pharmacies | Cross-sectional study | *Simulated client presenting on behalf of a friend suffering from pain on urinating and a urethral discharge for past 3-4 days (5 clients per shop- 300 encounters). *Semi-structured interview questionnaire to assess reported management of an STD client. | 1&2 |
| Chalker et al 2005*, Vietnam and Thailand | 68 private pharmacies in Vietnam, 78 private pharmacies in Thailand | Multi-component intervention study (regulatory enforcement, training, peer influence) evaluated using a cluster randomised controlled trial with a time series design. | *Simulated client for 2 scenarios (request to purchase antibiotic without a prescription, request to purchase steroid without a prescription). *4 rounds (a baseline and after 1 month of each of the 3 interventions). | 2 |
| Chua et al 2013, Malaysia | 10 (5 corporate, 5 independent) | Cross-sectional study | *Observation of interactions between pharmacist and customers for 4 days in each pharmacy. Data recorded according to structured data collection form. | 1 |
| Chuc and Tomson 1999, Vietnam | 2 pharmacies | Case study | *Observation (observed all encounters for 2 weeks) and interviews with customers. *Inventory and observation of operation. *Informal chats and in-depth interviews with staff regarding economic issues and good pharmacy practice. | 1&2 |
| Chuc et al 2001*, Vietnam | 60 private pharmacies | Cross-sectional study | *Simulated client presenting as the mother of a 3-5 year old at home with cough for 2 days (uncomplicated RTI). * Semi-structured interview questionnaire to assess reported management of RTI client. | 1&2 |
| Chuc et al 2002*, Vietnam | 68 registered private pharmacies | Multi-component intervention study (regulatory enforcement, training, peer influence) evaluated using a RCT with a time series design. | *Simulated client for 4 tracer conditions (upper RTI in a child, a STD, request to purchase an antibiotic without a prescription, request to purchase a steroid without a prescription). 4 rounds (a baseline and after 1 month of each of the 3 interventions). | 1&2 |
| Cong et al 1998 , Vietnam | 125 private sector drug vendors also known as alternative treatment providers (ATPs), including private doctors, private pharmacies, government and private drug outlets (breakdown not given) | Cross-sectional study | * Structured interviews with drug vendors. *Observation of storage, handling and sale of antimalarials. * Simulated patient presenting with 3-day long fever which subsides and keeps returning. | 1 |
| Dineshkumar et al 1995*, India | 32 retail pharmacy outlets | Cross-sectional study | *Observation and record of all sales for 2 complete days per pharmacy. *Structured interviews with patients who purchased medicine. | 1&2 |
| Dua 1994, India | 34 private pharmacies | Cross-sectional study | *Structured interview questionnaires for providers to elicit information on ownership and structure of business. *Structured interview questionnaires for clients to elicit demographic information and information concerning the pharmacy encounter. *Observation of sales (4-5 hrs per pharmacy). | 1&2 |
| Duong et al 1997b*, Vietnam | 25 private pharmacies | Cross-sectional study | *Interviews with customers based on structured questionnaire. | 1&2 |
| Duong et al 1997a*, Vietnam | 29 private pharmacies | Cross-sectional study | *Simulated client presenting as the mother of a child with diarrhoea. | 1 |
| Greenhalgh 1987, India | 12 private pharmacies | Cross-sectional study | *Observation of interactions in the pharmacy. | 1&2 |
| Hadi et al 2010, Indonesia | 75 pharmacies, 10 drug stores, 39 roadside kiosks | Cross-sectional study | *Simulated clients requesting first line antibiotics with and without a prescription. | 1 |
| Huda et al 2014, Bangaldesh | 331 private pharmacies | Cross-sectional study | *Simulated clients presenting with various scenarios relating to menstrual regulation/medical abortion. | 1 |
| Hussain and Ibrahim 2011*, Pakistan | 371 private pharmacies (96 type A, 186 type B and 66 type C) | Cross-sectional study | *Observation of 1113 patient-dispenser interactions (2 hours per pharmacy). | 1&2 |
| Hussain and Ibrahim 2012*, Pakistan | 371 private pharmacies (96 type A, 186 type B and 66 type C) | Cross-sectional study | *Simulated client presenting as older sibling of a 5 year old with diarrhoea. | 1 |
| Kafle 1998*, Nepal | 352 private pharmacies (baseline), 314 by second follow-up | Randomised controlled trial (combinations of training, mailed materials and audit feedback) | *Simulated clients presenting with various scenarios (parent of a child with diarrhoea, husband of a pregnant woman, parent of a child with a cough).  *Structured interview questionnaire for retailers to asses socioeconomic indicators at baseline and changes in knowledge. | 2 |
| Kafle et al 2013*, Nepal | 342 private pharmacies (baseline), 314 by second follow-up | Randomised controlled trial (combinations of training, mailed materials and audit feedback) | *Simulated clients presenting with various scenarios (parent of a child with diarrhoea, husband of a pregnant.woman, parent of a child with a cough).  *Structured interview questionnaire for retailers to asses socioeconomic indicators at baseline and changes in knowledge. | 2 |
| Kafle et al 1996, Nepal | 112 registered drug retailers | Cross-sectional study | *Simulated client presenting as the husband of a woman who 'is pale and looking weak'. *Structured interview questionnaire to assess knowledge of pregnancy problems. *Focus group discussions with drug retailers to explore motivations for current practice. | 1&2 |
| Kamat and Nichter 1998, India | 75 pharmacies (interviews); 6 pharmacies (observation and sales data) | Ethnography | *Semi-structured interviews with pharmacy owners.  *Participant observation for minimum of 10 days in 6 pharmacies. *Drug sales data for 3 full days in each of 6 pharmacies. *Exit interviews with customers who purchased a scheduled medicine without a prescription. *In-depth interviews with medical representatives. | 1&2 |
| Khan et al 2006*, Nepal | 37 private pharmacies | Cross-sectional study (presents 'after' results from a training programme) | *Simulated client presenting with history of urethral discharge (35 year old male) or vaginal discharge (inquiring for wife).  *Structured interview questionnaire to assess knowledge and reported practice. | 2 |
| Kotwani et al 2012, India | 40 pharmacists (retail, public sector, and office bearers of pharmacists' associations) | Exploratory single-site case study | *Focus group discussions. | 2 |
| Krishnaswamy et al 1985*, India | 33 retail pharmaceutical shops | Cross-sectional study | *Observation and record of all sales for 4 complete days per pharmacy. | 1 |
| Lansang 1990,  Phillipines | 59 private drug stores | Cross-sectional study | *Observation of transactions (monitored sales of all drugs for 3 days in small, single owned shops and for 2 days in larger branches of chain stores). *Structured interview questionnaire with all customers who asked for or bought antibiotic. | 1&2 |
| Larsson et al 2006*, Vietnam | 60 private pharmacies | Randomised control trial (baseline results only) | *Simulated patient requesting steroid tablets.  *Semi-structured questionnaire interview (mainly multiple choice, few open-ended questions) to gain info on reported practice. | 1&2 |
| Mac et al 2006, Vietnam | 33 public and private pharmacies (breakdown not given) | Cross-sectional study | *Simulated client requesting 10 units of the cheapest anti-epileptic drug (AED). *Structured survey questionnaire to establish a list of AEDs available and their prices. | 1&2 |
| Mamun et al 2006, Bangladesh | 5 private medicine-dispensing shop (main focus of study on rural medical practitioners) | Cross-sectional study | *Interviews with drug sellers. *Interviews with customers. *Reviewed sale books of shops. *Observation of medicine sales. | 1&2 |
| Minh et al 2013*, Vietnam | 220 private pharmacies | Before and after intervention study (training) | *Structured questionnaire with pharmacy staff (281) recording knowledge and reported practice for diarrhoea management.  *Simulated client seeking advice for 14 month old child with diarrhoea (baseline and 6-months post intervention). | 1&2 |
| Mondal et al 1994, India | 30 private pharmacies | Cross-sectional study | *Simulated client presenting as the father of a 2 year old boy with diarrhoea. | 1 |
| Nakajima et al 2010, Mongolia | 250 private pharmacies | Cross-sectional study | *Structured interview questionnaire for pharmacy workers mainly focussed on antibiotic medicines. *Structured interview questionnaire customers to elicit demographic information and details of medicine purchases. | 1 |
| Nga et al 2014, Vietnam | 30 private pharmacies | Cross-sectional study | *Observation of all drug sales at each pharmacy for 3 days. *Semi-structured questionnaire for pharmacy staff focussing on antibiotic sales. *In-depth interviews and focus group discussions with pharmacy staff regarding perceptions of factors affecting inappropriate antibiotic dispensing. | 1&2 |
| Ngo et al 2012, Vietnam | 100 registered private pharmacies | Cross-sectional study | *Structured interview questionnaire with pharmacy staff to assess knowledge of medical abortion. *Simulated clients presenting as either a young female seeking medical abortion or a young male seeking abortifacients for his girlfriend. | 1 |
| Pham et al 2013*, Vietnam | 220 private pharmacies | Before and after intervention study (training) | *Structured questionnaire with pharmacy staff recording knowledge and reported practice for diarrhoea management. *Simulated client seeking advice for 14 month old child with diarrhoea (baseline and 6-months post intervention). | 2 |
| Podhipak et al 1993, Thailand | 240 private pharmacies. 120 type I and 60 type II pharmacies in Intervention area. 60 of each type in control area | Before and after intervention study (training) | *Simulated clients presenting as mothers of children with watery diarrhoea and dysentery. | 1&2 |
| Puspitasari et al 2011,Indonesia | 105 private pharmacies | Cross-sectional study | *Simulated clients presenting with various scenarios (request for 10 tablets of ciprofloxacin; request for 2 capsules of tetracycline; prescription for paediatric amoxicillin dry syrup). | 1 |
| Qidwai et al 2006, Pakistan | 6 private drug sellers | Before and after intervention study (training) | *Structured questionnaire to obtain shop and drug seller characteristics. *Simulated clients presenting with various scenarios of diarrhoea. | 1&2 |
| Rahman et al 2000, Bangladesh | 157 private pharmacies | Cross-sectional study | *Self-administered questionnaires for medicine sellers exploring knowledge and reported practices relating to STD/AIDS management. *Simulated clients presenting with an STD. | 1 |
| Ratanajamit et al 2001*, Thailand | 30 pharmacist-owned and 30 non-pharmacist owned drug stores | Baseline results of randomised controlled trial | *Structured interview questionnaire to measure knowledge of oral and emergency contraception. *Simulated clients requesting oral contraception and emergency contraception (on different occasions) | 1&2 |
| Ratanajamit et al 2002*, Thailand | 30 pharmacist-owned and 30 non-pharmacist owned drug stores | Randomised controlled trial (educational intervention) | *Simulated client presenting as college student who experienced unprotected mid-cycle sex 24 hours earlier. *Structured interview questionnaire to assess knowledge. | 1&2 |
| Rathnakar et al 2012, India | 60 private pharmacies | Cross-sectional study | *Simulated clients presenting with various scenarios (URTI, acute bronchitis, diarrhoea accompanied with pain and fever). | 1 |
| Ross-Degnan et al 1996, Indonesia | 87 private pharmacies (43 intervention, 44 control) | Randomised control trial (educational intervention) | *Structured interview questionnaires to assess knowledge of diarrhoea management. *Simulated clients presenting as the mother of a child under 5 with diarrhoea (before and after intervention). *Focus group discussions with pharmacy staff to understand factors underlying treatment behaviours. | 1&2 |
| Roy J 1997, Bangladesh | Sample of village 'doctors' (pally chikitshak/pharmacy salespersons). Numbers not given. | Cross-sectional study | *Interviews.  *Direct observation of practice. | 1&2 |
| Saencharoen and Lerkiatbundit 2013*, Thailand | 142 private pharmacies | Cross-sectional study | *Simulated clients presenting with mild or moderate migraine. *Structured interview questionnaire to assess knowledge regarding migraine management. | 1&2 |
| Saengcharoen and Lerkiatbundit 2010*, Thailand | 115 private pharmacies (96 type I and 29 type II) | Cross-sectional study | *Simulated client presenting as the aunt of a 4 year old with watery diarrhoea. *Written questionnaire collecting data on demographics and knowledge and attitudes towards diarrhoea management. | 1&2 |
| Saradamma et al, India | 11 private pharmacies | Cross-sectional study | *4 hours of observation per pharmacy. *Structured interviews with customers who purchased antibiotics. | 1 |
| Seeberg 2012, India | 20 private practitioners and chemists | Ethnography | *In-depth interviews and clinical observations over an 18-month period. | 1&2 |
| Stenson et al 2001a*, Lao PDR | 106 private pharmacies (mostly class 3) | Cross-sectional study | *Structured interviews with the drug sellers. *Facility-specific indicator survey of the pharmacies.  *Semi-structured exit interviews with customers outside the pharmacies. *Inspection of drug purchases. *Recording of prices of sample drugs. *Structured interviews with district drug inspectors. | 1 |
| Stenson et al 2001b*, Lao PDR | 92 private pharmacies - mostly class 3 (46 regular intervention, 46 active intervention) | Randomised controlled trial (regulatory intervention) | *Structured interview with drug seller and inspection of premises to assess facility-specific indicators. *Semi-structured exit interviews and review of medicines purchased to obtain dispensing indicators. | 2 |
| Syhakhang et al 2001*, Lao PDR | 92 private pharmacies (mostly class 3) and 13 public pharmacies | Cross-sectional study | *Structured interviews with the drug sellers. *Facility-specific indicator survey of the pharmacies.  *Semi-structured exit interviews with customers outside the pharmacies. *Inspection of drug purchases. | 1 |
| Thamlikitkul 1988, Thailand | 40 1st class drug stores | Cross-sectional study | *Simulated clients presenting with various conditions (urethral discharge in adult male; cut wound in 4 year old, impetigo in 2 year old; acute watery diarrhoea in 6 month old; acute watery diarrhoea in adult; fever with sore throat in adult; fever, rhinorrhoea and cough in a 2 month old; fever with rhinorrhoea in adult; acute dysuria in adult female. | 1 |
| Tomson and Sterky 1986, Bangladesh, Sri Lanka and Yemen Arab Republic | 25 private pharmacies in each country setting | Cross-sectional study | *Simulated client (Caucasian man) presenting as the father of a 11 month old with diarrhoea of 3 days duration. | 1&2 |
| Tuladhar et al 1998, Nepal | 160 private pharmacies | Before and after intervention study (training) | *Simulated clients reporting urethral discharge. | 1&2 |
| Van Sickle 2006, India | 52 private pharmacies | Cross-sectional study | *Simulated client presenting with symptoms of asthma for preceding 2-3 weeks in themselves or a child (aged 10 or 14). | 1&2 |
| Vu et al 2012, Vietnam | 138 private pharmacies | Cross-sectional study | *Simulated client presenting with symptoms of tuberculosis and request for anti-TB drugs. *Structured interview questionnaire to record information on pharmacy, provider, management of a fictious case and knowledge of the national tuberculosis programme. | 1&2 |
| Wachter et al 1999, Nepal | 100 private pharmacies listed in Nepal Chemists' and Druggists' Association | Cross-sectional study | *Simulated clients presented two scenarios (a case of cystitis in 25 year old male, 5 year old son with loose stools for 1 day). | 1 |
| Wolffers 1987, Sri Lanka | 28 private pharmacies | Cross-sectional study | *Simulated clients requesting 2 tablets of tetracycline. | 1 |

*One of several papers from the same research study. The following sets of papers appear to come from the same research projects:

- Stenson et al 2001a, Stenson et al 2001b, Syhakhang et al 2001

- Saencharoen and Lerkiatbundit 2013, Saencharoen and Lerkiatbundit 2010

- Ratanajamit et al 2001, Ratanajamit et al 2002

- Pham et al 2013, Minh et al 2013

- Krishnaswamy et al 1985, Dineshkumar et al 1995

- Khan et al 2006, Bista et al 2002

- Larsson et al 2006, Chalker et al 2000, Chalker et al 2005, Chuc et al 2001, Chuc et al 2002

- Kafle 1998, Kafle et al 2013

- Hussain and Ibrahim 2011, Hussain and Ibrahim 2012

- Duong et al 1997a, Duong et al 1997b
